# High rate of complications after corrective midfoot/subtalar arthrodesis and Achilles tendon lengthening in Charcot arthropathy type Sanders 2 and 3

**DOI:** 10.1007/s00264-022-05567-y

**Published:** 2022-09-22

**Authors:** Markus Regauer, Veronika Grasegger, Julian Fürmetz, Adrian Calvacanti Kussmaul, Wolfgang Böcker, Christian Ehrnthaller

**Affiliations:** 1 Department of Orthopaedics and Trauma Surgery, Musculoskeletal University Center Munich (MUM), University Hospital, LMU Munich, Marchioninistr. 15, 81377 Munich, Germany; 2 Sportortho Rosenheim, Rosenheim, Germany; 3 Department of Trauma Surgery, BG Trauma Center MurnauMurnau Am Staffelsee, Murnau, Germany

**Keywords:** Achilles tendon lengthening, Charcot arthropathy, Corrective midfoot arthrodesis, Foot arch collapse, Hindfoot arthrodesis

## Abstract

**Purpose:**

Corrective midfoot resection arthrodesis is the standard treatment of Charcot arthropathy type Sanders 2 and 3 with severe dislocation. In order to critically evaluate the effect of surgical correction, a retrospective analysis of our patient cohort was performed. Hereby, special emphasis was set on the analysis of the pre- and post-operative equinus position of the hindfoot.

**Methods:**

Retrospectively, all patients (*n* = 82) after midfoot resection arthrodesis in Charcot type Sanders 2 or 3 were included. Complications were recorded, and the mean complication-free interval was calculated. Additionally, the calcaneal pitch as well as Meary’s angle were measured pre- and post-operatively and in case of complications.

**Results:**

Overall complication rate was 89%. Revision surgery was necessary in 46% of all patients. The mean complication-free interval was 285 days (0–1560 days). Calcaneal pitch and Meary’s angle significantly improved after operation but returned to pre-operative values after onset of complications. Achilles tendon lengthening showed no significant effects on the mean complication-free interval.

**Conclusion:**

Operative treatment of Charcot arthropathy remains a surgical challenge with high complication rates. Surgical correction of equinus position has been highlighted for successful treatment but was not able to prevent complications in this study, which is demonstrated by the recurrent decrease of the calcaneal pitch in cases of reoperation. Therefore, as a conclusion of our results, our treatment algorithm changed towards primarily addressing the equinus malpositioning of the hindfoot by corrective arthrodesis of the hindfoot.

## Introduction

Charcot arthropathy is defined as a primarily non-infectious, chronic-progressive degeneration and destruction of the skeletal parts of the foot and ankle. It is also known as Charcot neuropathy, diabetic neuropathic osteoarthropathy, or neuropathic osteoarthropathy [2, 29].

Although the exact pathophysiological path leading to the characteristic destructions of the foot is not completely elucidated, two main theories are present with a neurotraumatic and a neurovascular approach. The neurotraumatic theory describes recurrent micro-traumata being responsible for a chronic inflammatory process in the affected tissues, whereas the neurovascular theory imposes vascular shunting due to alterations in the sympathetic nervous system with consecutively increased blood flow resulting in osteopenia [6, 36, 37]. In both cases, collapse of the physiological static of the foot is evident due to massive bony destructions and joint dislocations. Currently, the American Diabetes Association favours a hybrid theory [31].

Clinically, in its early stages, Charcot arthropathy presents with mostly painless swelling, warmth, and erythema of the feet and lower ankles [25]. The most common radiological classification for disease stage is the Eichenholtz classification [14]. Based on the affected structures, the Sanders-Frykberg classification is largely accepted [26]. Here, stage 2 and 3 represent the most affected midfoot area including the Chopart and Lisfranc joints. Due to the osteolytic destructive processes in the affected bones and joints, fractures and dislocations take place, which are not noticed by the patients due to the underlying neuropathic disease. Mainly, the pathological processes are prevalent in the midfoot section [27, 33]. In the late stages, flattening of the foot arch and plantar ulcerations are predominant, possibly leading to severe septic complications like major amputation of the involved leg [19, 25, 33].

In early stages, conservative treatment with immobilization in a total contact cast is known to be the gold standard, but double upright foot–ankle orthoses or custom-made C.R.O.W (Charcot Restraint Orthotic Walker) boots have been postulated as to be sufficient enough for successful conservative treatment [13, 24].

In the late stages after collapse of the medial and longitudinal foot arch with imminent ulcerations of the foot sole, surgical treatment is abundant.

As the pathological fractures mainly occur in the midfoot section [27, 33], surgical procedures concentrate to this region. Angular stable plate fixation after resection of all avital bone fragments seems to be the most dominant type of fixation, but various intraosseous devices have been developed, such as the midfoot fusion bolt. Making stabilization possible by minimally invasive stab incision, this type of osteosynthesis has gained a lot of interest [23]. Sadly, several implant failures showed that even those solid bolts do not provide enough stability to maintain a long-lasting stabilization [4, 16]. Therefore, combination with other implants has been postulated to be necessary [12, 16].

The role of the pathological hindfoot inclination towards an equinus position has gained more and more interest. Although biomechanical studies have shown that hindfoot equinus significantly increases plantar pressure and that patients suffering from Charcot neuropathy exhibit increased plantar pressures during gait cycle, the definite proof for its pathophysiologic role is still missing [5, 17]. Nevertheless, treatment of hindfoot equinus is generally accepted to reduce plantar pressure and promote healing of plantar ulcerations with and without Charcot neuropathy. Therefore, in almost all current treatment regimes, correction of the pathological equinus position of the hindfoot represents one of the key elements when surgical reconstruction of the collapsed bony structures is planned [1, 3, 12].

Current surgical treatment protocols are mainly based on the superconstruct theory by Sammarco et al. [32] describing the following factors being necessary for successful treatment:

- Extension of fusion to the area of vital bone
- Utilization of the strongest possible fixation device
- Bone resection as needed to reduce the deformation accepting shortening of the extremity
- Application of fixation devices in a position of maximal mechanical advantage

While no order of significance was implied in the original publication, the application sequence leaves room for individual treatment concepts. Whereas some surgeons prefer single-stage procedures with Achilles tendon lengthening and internal fixation devices after corrective arthrodesis as used by the authors, multi-stage procedures are favored by others. Here, the first stage mainly consists of Achilles tendon lengthening, application of an external fixator, and debridement of any infected bone. In the second stage, definitive reconstruction with internal fixation using the superconstruct theory is applied [12, 34].

The aim of this study was to show results of single-stage corrective midfoot and subtalar arthrodesis in a cohort of Charcot patients and to be able to improve future treatment strategies.

## Patients and methods

### Patients

All patients with Charcot arthropathy type Sanders 2 and 3 undergoing surgical corrective arthrodesis of the subtalar, Chopart, and Lisfranc joint line at a single level 1 trauma centre over a 12-year time period were retrospectively included in the study.

#### Inclusion criteria

- Charcot arthropathy type Sanders 2 and 3
- Surgical single-stage corrective midfoot as well as subtalar arthrodesis

#### Exclusion criteria

- Infected tissues/open plantar wounds
- Acute inflammatory disease stage
- Multi-stage corrective arthrodesis
- Arthrodesis of the upper ankle joint

### Surgical treatment

All patients underwent a single-stage corrective arthrodesis of the affected bones and joints (Lisfranc, Chopart, subtalar) according to the Sammarco superconstruct theory. For Lisfranc or Chopart arthrodesis, a standard medial and lateral approach was used. For subtalar pathologies, an open lateral subtalar approach was performed. Whereas subtalar pathologies were addressed using screws, Chopart as well as Lisfranc arthrodesis was achieved using plates from one single manufacturer (DePuySynthes) with the use of locking or non-locking screws according to the clinical situation. In case of a pre-operatively pathologic equinus position of the hindfoot after assessment of the Silfverskjöld test, either a gastrocnemius recession and/or a z-lengthening of the Achilles tendon was performed. The extent of lengthening was adjusted intra-operatively after definitive fixation of the corrective arthrodesis. The surgical procedures were performed by four different senior foot and ankle surgeons. Post-operatively, all patients received a custom-made C.R.O.W (Charcot Restraint Orthotic Walker) boot completely unloading the foot for 12 weeks. After this period, every patient received a custom-made orthotic shoe with insoles for an optimized plantar pressure distribution and minimized risk for future complications.

### Follow-up

After surgery, all patients were routinely followed up after six weeks, after three and six months, and after one year. Besides clinical examination, at each follow-up visit, x-rays were taken in two planes. Weight-bearing x-rays have been performed after confirmation of complete healing of the arthrodesis and whenever possible regarding the patients physical abilities.

### Data analysis

Data collection was performed retrospectively by review of patients’ charts and analysis of the electronic clinical documentation system.

Patient-driven parameters included age, sex, height, weight, ASA classification, diabetes type, HbA1c, smoking and alcohol abuse, peripheral vascular disease, documented incompliance, and medical immunosuppression. Regarding the performed surgical therapy, the following parameters were recorded: Sanders classification, date of surgery, localization of arthrodesis, implants applicated, additional procedures like Achilles tendon lengthening, hospitalization time, and complication-free time interval.

### Angle measurement

Objective review of the patient’s x-rays was performed by measurement of the Calcaneal pitch as well as Meary’s angle (Fig. 4). Measurements were recorded for each case by the available x-rays before and immediately after index operation and in case of re-operation. Whenever possible and available, weight-bearing x-rays were used for angle measurement.

### Complications

Complications were grossly classified into mechanical (implant-associated) and septic (infection-driven) complications. Necessity for revision surgery distinguished between major and minor complications. As some patients suffered from more than one complication, complications were distinguished between a first primary complication and additional secondary complications. In Table 1, some examples for classification are shown.

**Table 1:** Examples for possible complications and their classification

Type of complication	Minor complication (no need for revision surgery)	Major complication (revision surgery necessary)
Mechanical	Implant failure, no instability	Nonunion, implant failure with instability
Septic	Wound healing complication, erysipel	Osteitis, osteomyelitis, deep wound infection

### Statistics

Data collection was performed with pseudonymized data recorded in Excel (Microsoft Corporation, Redmond, WA, USA). IBM SPSS Statistics, Version 26 (IBM Corp. Released 2016, Armonk, NY) was used for statistical analysis. Data are reported as either mean ± 2 time standard error of the mean (SEM). First, descriptive statistics was performed. Afterwards, testing for normal distribution was performed using a Kolmogorov–Smirnov Test. Consecutively, a Student’s t-test, ANOVA, or Pearson correlation was performed depending on the data. A *p*-value < 0.05 was regarded to be statistically significant.

## Results

### Descriptive analysis

In total, 84 patients were enrolled in the study. Sixty-three patients (75%) were male, and 21 were female (25%). The youngest patient was 37 and the oldest 79 years old. The mean age was 59 ± nine years. In 38 patients (45.2%), the left foot and in 46 patients (54.8%) the right side were affected. The mean height was 174.9 ± 8.9 cm, and the mean body weight measured 91.7 ± 20.3 kg. The calculated mean body mass index was 29.4 ± 5.6 ranging from 18.8 to 50.7. Mean hospitalization time was 16.2 ± 14.3 days. The minimum hospital stay was five days, while the longest was 96 days from the index operation. The mean pre-operative HbA1c was 7.2 ± 1.8% with the minimum being 4.4% and the maximum 13.6%. Further patient characteristics and risk factors are displayed in Tables 2 and 3.

**Table 2:** Patient characteristics and risk factors in total numbers and percent. *ASA* American Society of Anesthesiologists’ classification

	ASA					Diabetes			Dialysis		Smoking		Alcohol	
	I	II	III	IV	V	Type I	Type II	No	Yes	No	Yes	No	Yes	No
Total number	0	36	45	1	0	10	64	9	4	77	51	31	35	46
%	0	43.9	54.9	1.2	0	12	77.1	10.8	4.9	95.1	62.2	37.8	43.2	56.8

**Table 3:** Patient characteristics and risk factors in total numbers and percentage. *PAD* peripheral arterial disease stages 1–4 according to Fontaine

	Immunosuppression		Compliance		PAD					
	Yes	No	Yes	No	No	1	2	3	4	Unknown
Total number	4	78	71	12	57	4	2	1	3	16
%	4.9	95.1	85.5	14.5	68.7	4.8	2.4	1.2	3.6	19.3

### Surgical treatment

Implants were chosen according to the individual pathology and affected bones and joints. Pathology of the medial column and the first three metatarsals are referred to as medial plates and screws. Pathology of the subtalar joint as well as the calcaneo-cuboidal joint and pathology of the fourth and fifth metatarsals is referred to as lateral. Details on the used implants are displayed in Table 4.

**Table 4:** Implants used for correction of Charcot deformation in total numbers and percentage

	Implants					
	Medial plate	Lateral plate	Medial and lateral plates	Medial plate and lateral screws	Lateral plate and screws	Medial and lateral plates and screws
Total number	11	0	42	12	2	5
%	13.4	0	51.2	14.6	2.4	6.1

### Achilles tendon lengthening

In 68.8% of all patients, no Achilles tendon lengthening was performed. While in 28.7% Achilles tendon lengthening or gastrocnemius recession has been performed during the index operation, surgical lengthening prior and after the index operation has been performed in one patient each.

### Complications

#### Primary complications

**Fig. 1:**
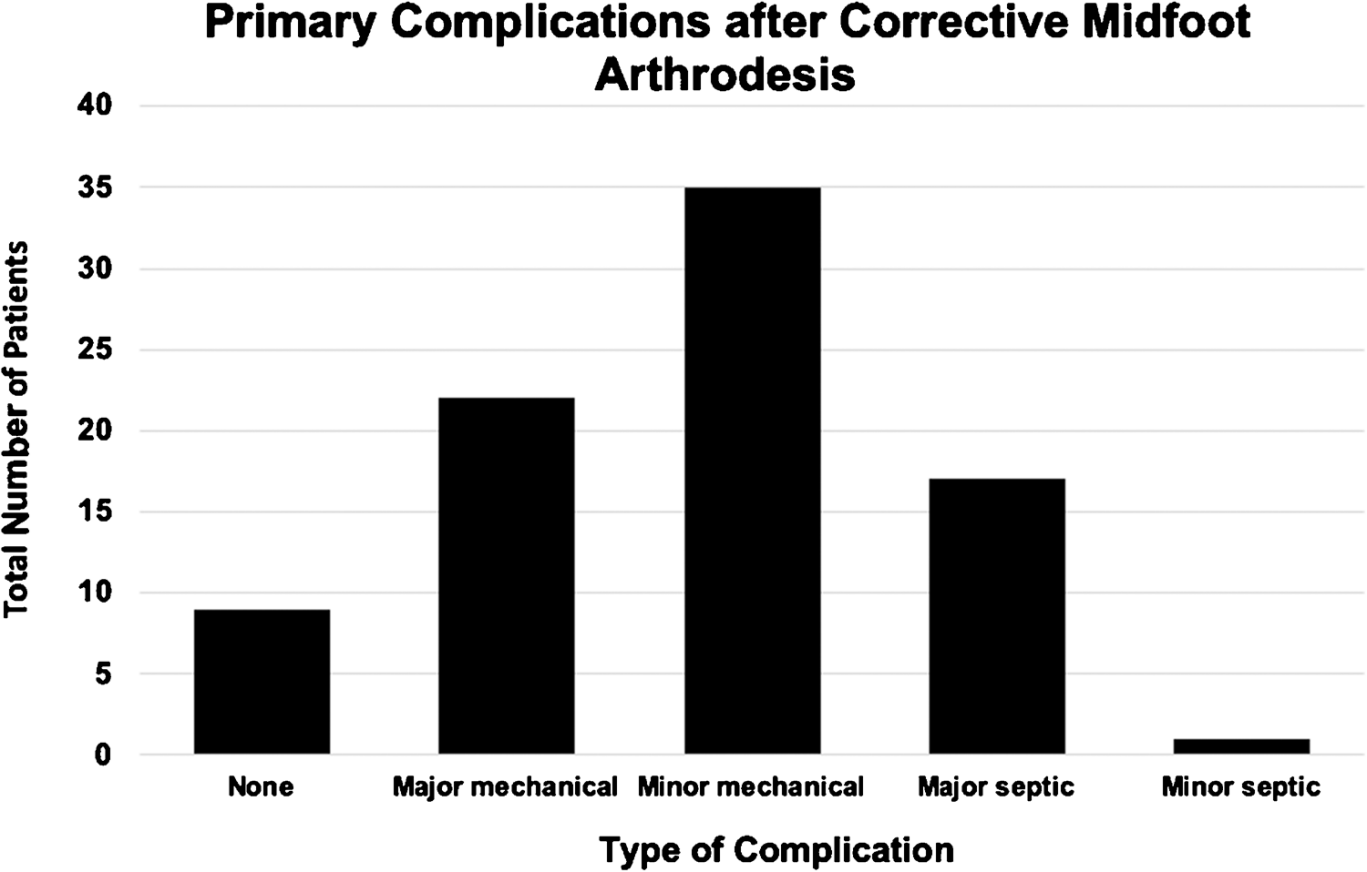
Primary complications after corrective midfoot arthrodesis in total number of patients regarding different types of complications

While only nine patients (11%) stayed without complications, 75 patients (89%) showed at least one complication. Thirty-nine patients suffered from a major complication with need for revision surgery (Fig. 1).

**Fig. 2:**
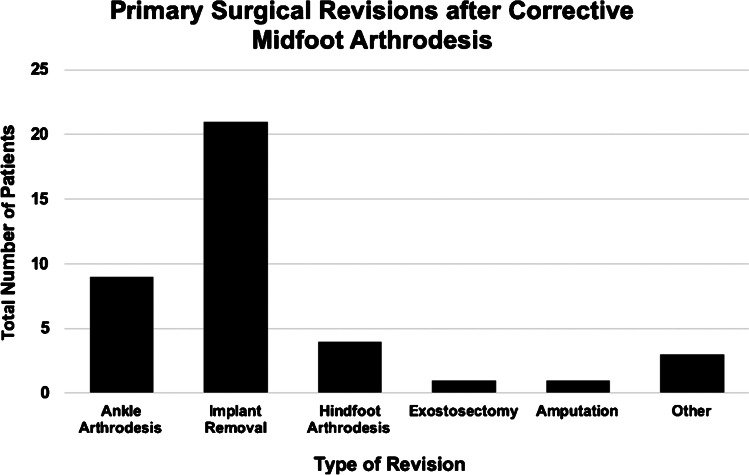
Primary surgical revisions after corrective midfoot arthrodesis in total number of patients regarding different types of revision surgery

Overall, mechanical complications accounted for 68% of all cases. In cases of necessary surgical revision, 48% were treated by implant removal followed by arthrodesis of the ankle joint (23%) or hindfoot arthrodesis (10%). Amputation was only necessary in one patient (Fig. 2).

Complication-free interval was also documented. Here, a mean onset of the primary complication was documented after 285 days with a minimum of 0 and a maximum of 1560 days.

#### Secondary complications

Seventeen out of 75 patients (23%) with a primary complication suffered from another complication during the follow-up period.

Here, major septic complications were predominant, accounting for 13 out of 17 cases (76%), whereas only one patient had major mechanical complication.

**Fig. 3:**
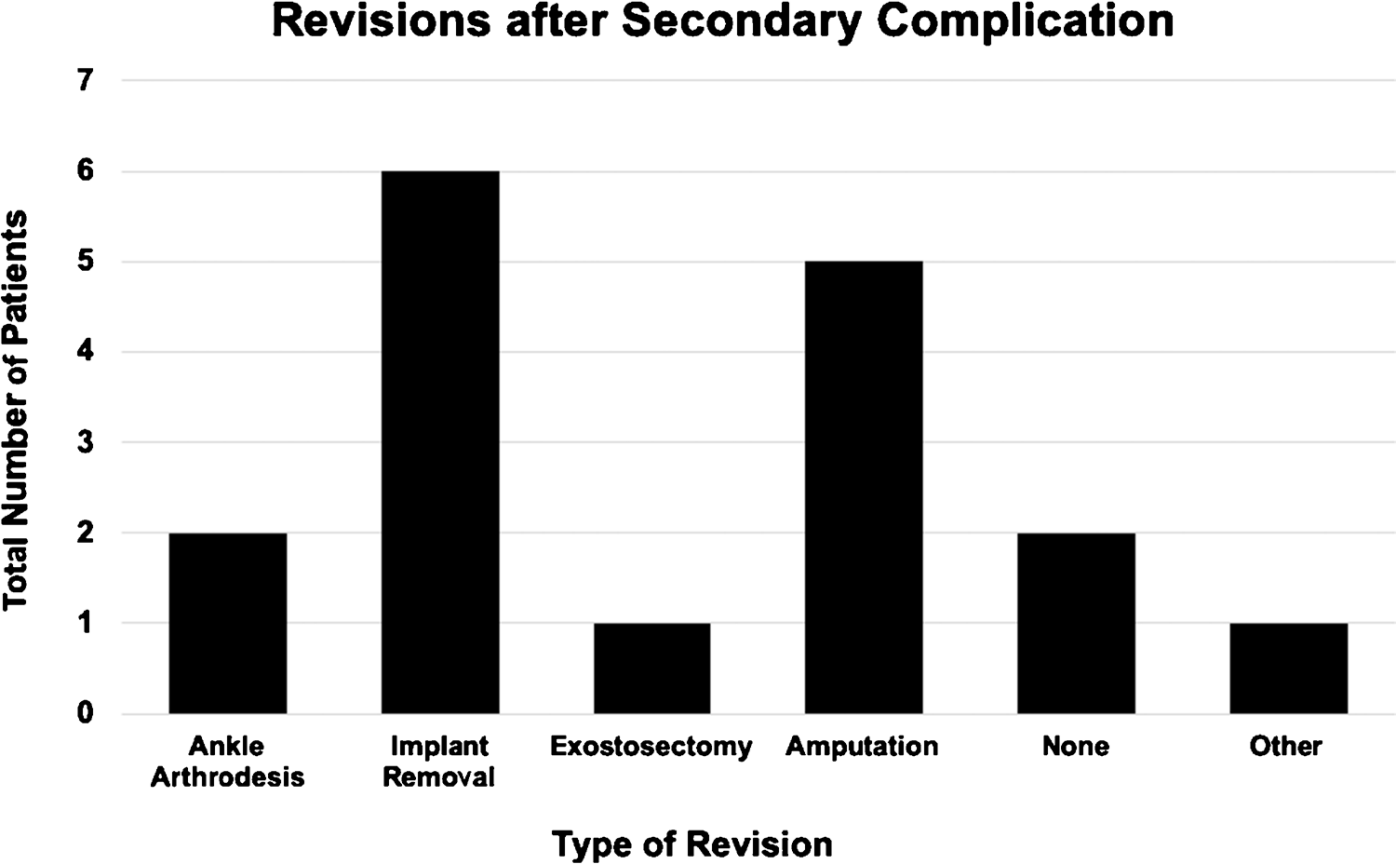
Surgical revisions after corrective midfoot arthrodesis in total number of patients regarding different types of revision surgery

While again most patients could be treated by implant removal (35%), the rate of necessary amputations increased towards 29% followed by ankle arthrodesis with 12% (Fig. 3).

### Angle measurements

**Fig. 4:**
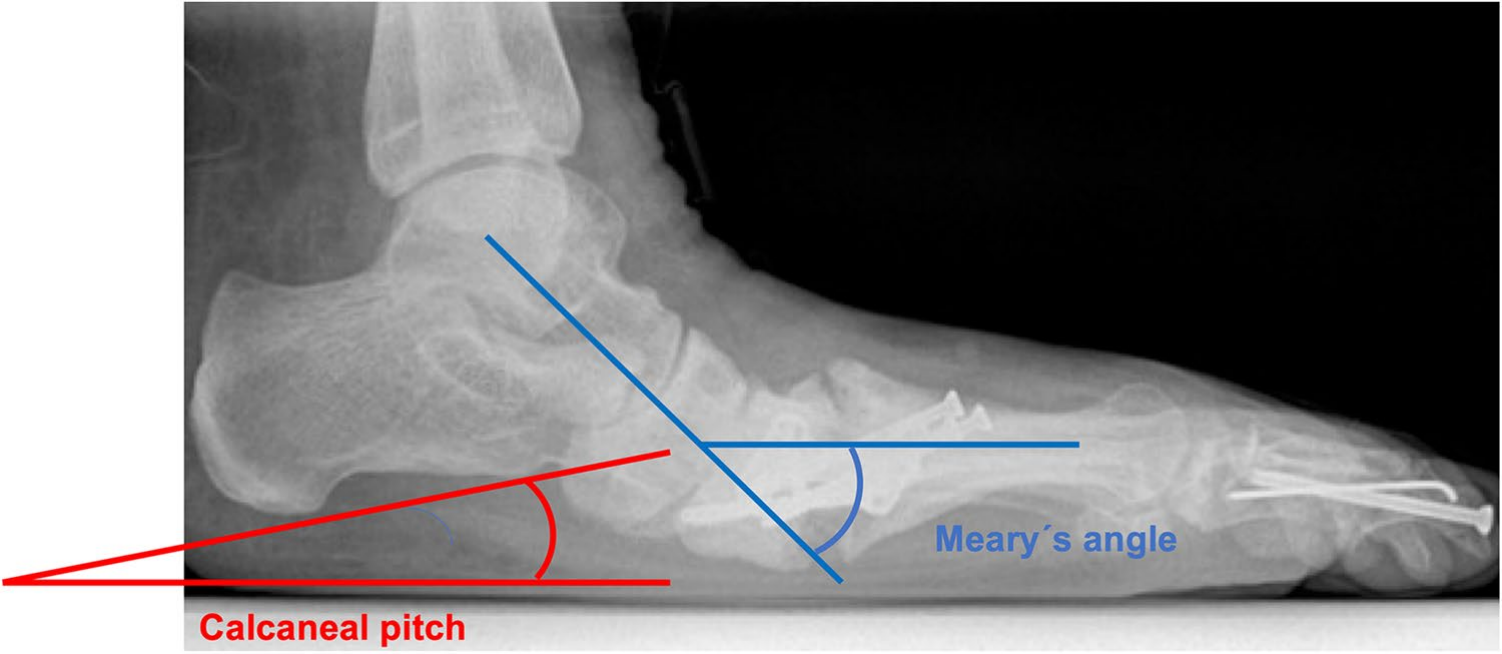
Illustrative example for the measurement of calcaneal pitch (red) and Meary’s angle (blue)

**Fig. 5:**
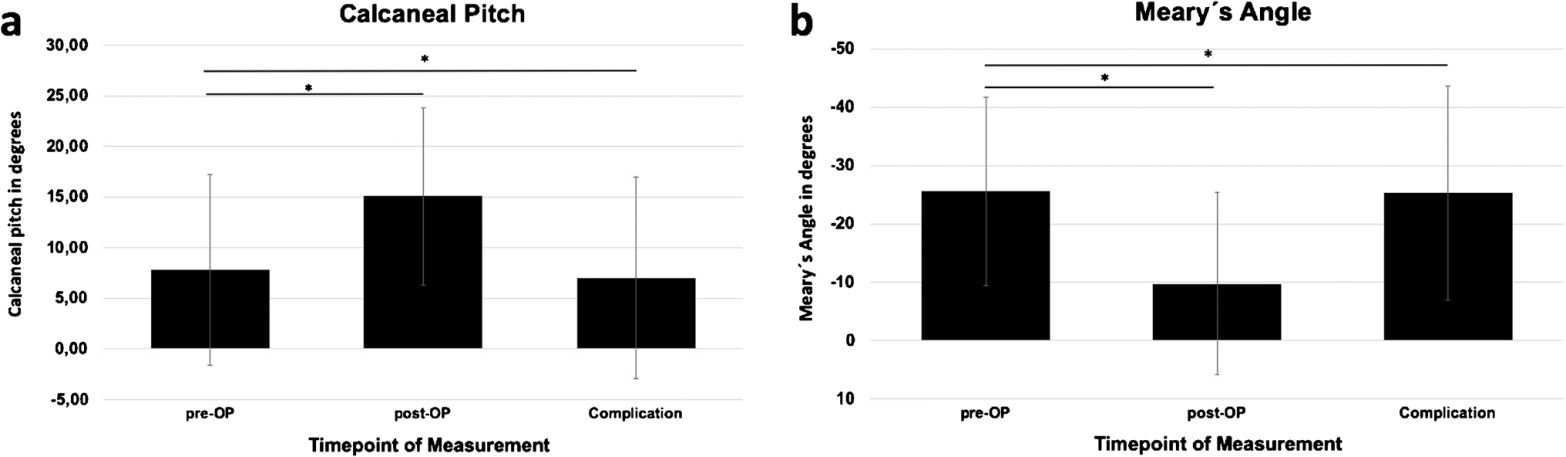
Measurements of calcaneal pitch (**a**) and Meary’s angle (**b**) in degrees at different points in time (*p < 0.05)

Statistical evaluation of the time-dependent angle measurements showed significant differences for both calcaneal pitch (inclination) and Meary’s angle. In detail, for both types of angles, differences between pre- and post-OP angles as well as differences between post-OP measurements and measurements at onset of complications were statistically significant (Figs. 4 and 5).

### Correlation of risk factors with complication rate

The following parameters were analyzed and showed no significant correlation with the complication rates:

- Sex (*p* = 0.104)
- ASA classification (*p* = 0.088)
- BMI (*p* = 0.272)
- Diabetes mellitus/HbA1c (*p* = 0.865/0.770)
- Alcohol or nicotine abuse (*p* = 0.98/0.746)
- Compliance (*p* = 0.241)
- Achilles tendon lengthening (*p* = 0.216)

## Discussion

Surgical treatment of Charcot arthropathy still remains a great challenge, especially when complicated by a breakdown of the foot arch with imminent or already present plantar ulcerations. Therefore, various treatment regimens exist, but evidence for the best clinical strategy is still missing [1, 3, 9, 11, 12].

In this study, we demonstrated that after a single-step surgical deformity correction of the midfoot, a total of 87% of patients suffered from at least one complication, and 45% even needed revision surgery. This complication rate is in accordance with the pertinent literature where also high revision rates have been reported so far [13, 18, 23, 30].

### Novel surgical implants

The main driver for implant-associated failures seems to be the mechanical overload of the bone structures in proportion to their limited healing capacity [13]. During the last years, novel implants have been developed, with the ultimate goal to reduce implant-associated failure rates [23]. The Midfoot Fusion Bolt, for example, has been developed as a minimal invasive tool for fixation of dislocated bone structures, but even though being made of solid titanium, the implant seems not to be able to permanently stabilize the affected bone structures. Current studies were able to demonstrate that these novel implants produce a high number of failures [4, 16].

### Equinus position of the hindfoot

Another problem which has been identified and highlighted during the last years is the equinus position of the hindfoot [10, 20]. Besides missing proprioception and a consecutive feedback loop in polyneuropathic patients while weight-bearing, shortening of the Achilles tendon complex creates mechanical forces working against the direction of surgically achieved reduction, ultimately leading to failure of the implant [22, 30].

Therefore, current treatment protocols highlight the importance of surgical treatment of the hindfoot equinus position by lengthening the Achilles tendon either open or minimally invasively [1, 3, 10, 12, 28, 30].

In the present work, Achilles tendon lengthening was performed in case of a pathologic hindfoot equinus position in the clinical examination before surgery. However, Achilles tendon lengthening did not protect patients from suffering complications as this procedure was demonstrated not to have a significant effect on the mean complication free interval.

**Fig. 6:**
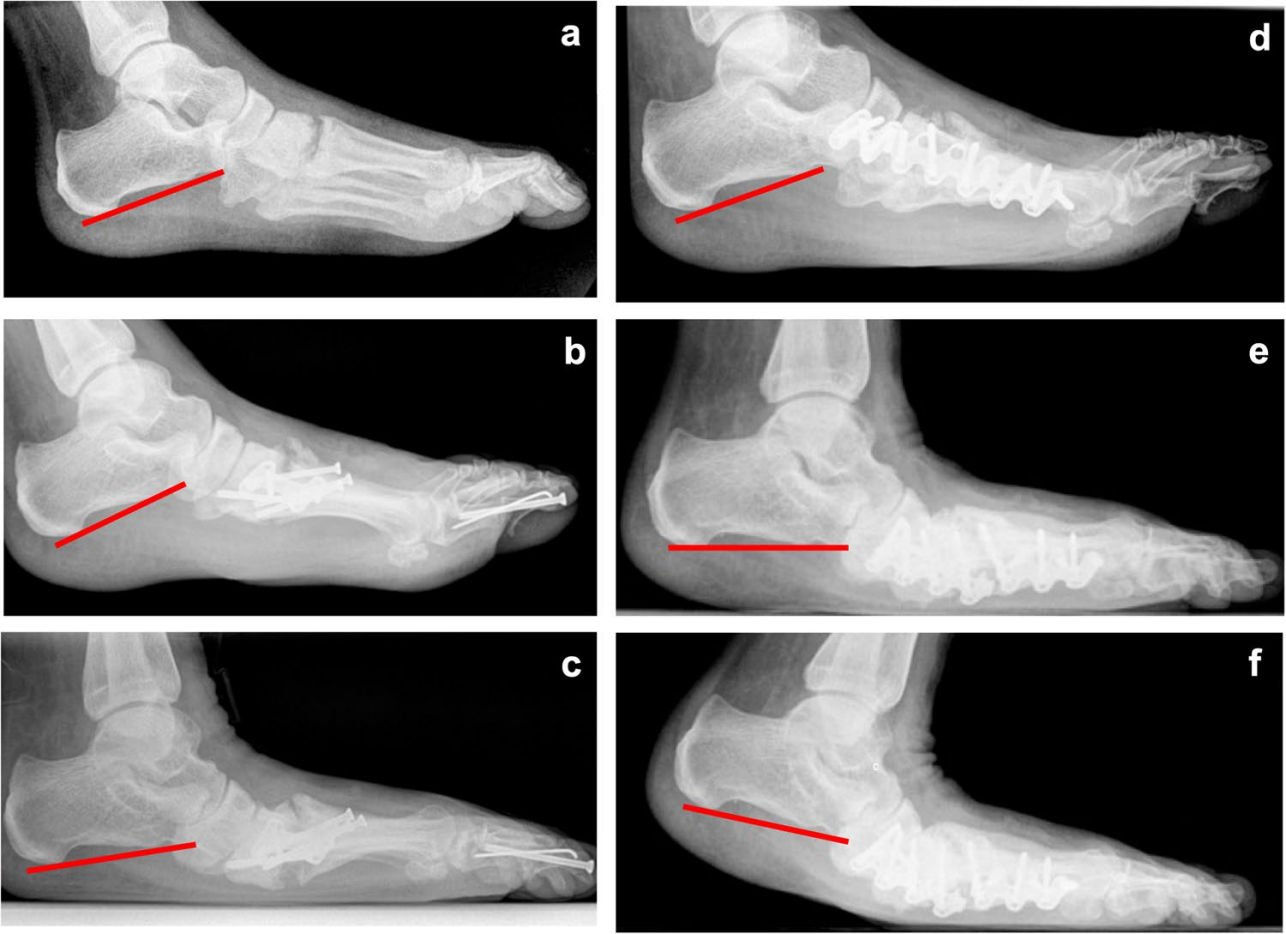
Example of a case treated with corrective midfoot arthrodesis in a 48-year-old diabetic patient with Charcot arthropathy treated with non-locking implants and consecutive implant failure (**a**–**c**). After failure, locking implants were used (**d**–**f**). However, also these locking implants have not been able to permanently maintain reduction, and recurrent implant failure occurred

Although effective correction of the equinus position was achieved post-operatively, at the time of complication, the calcaneus pitch significantly returned to pathologic values like before initial surgical correction (Fig. 6). Therefore, it seems that even though surgical correction had been performed, the underlying pathophysiological processes are advancing in the further course of the disease, ultimately forcing the hindfoot back into an equinus position leading to consecutive failure of the midfoot osteosynthesis [34].

### Single-stage or multi-stage surgical protocol

In contrast to our study, authors report on satisfactory results in staged protocols after Achilles tendon lengthening, preliminary fixation in an external fixator after debridement, and consecutive internal fixation with plates and screws according to the “superconstruct” theory [12, 34, 35].

It is tempting to speculate that one of the key factors in those staged treatment regimens is the longer time period of initial external fixation before definitive surgery. All published staged treatment protocols inherit a prolonged hospitalization and treatment time until complete weight-bearing is achieved. Although multi-stage procedures are sometimes necessary, e.g., in cases of infected plantar ulcerations, long treatment regimens incorporate various disadvantages especially for patients without ulcerations even though complication rates still remain high.

### Hindfoot arthrodesis

Recently, several studies were able to show a successful surgical treatment of Charcot arthropathy of the ankle and hindfoot region by means of tibio-talo-calcaneal arthrodesis [7, 15]. A meta-analysis recently compared the different implant types for surgical treatment and concluded that arthrodesis via a hindfoot intramedullary nail is the treatment of choice when the hindfoot is involved. Only in cases of plantar ulceration, the external fixator is described as a sufficient option [8].

Following the principles of the superconstruct theory by Sammarco et al., recent studies showed significantly improved outcomes when the principle of implant-extension to healthy bone in order to maximize stability was applied to the non-affected parts of the hindfoot [21].

Reflecting the favourable results of hindfoot arthrodesis using a tibio-talo-calcaneal nail in hindfoot Charcot arthropathy and the increasing extension of midfoot Charcot arthrodesis to the subtalar joint, the question arises if surgical reconstruction of midfoot Charcot arthropathy should focus more on the beginning of the pathologic cascade, namely the equinus position of the hindfoot. As Achilles tendon lengthening alone is obviously not capable for permanent reconstruction of the equinus deformity, the consequent development of the superconstruct theory would include arthrodesis of the non-affected ankle joint, creating a permanent reconstruction of the equinus and achievement of maximal stability.

### Limitations

The main limitation of our study is the missing of a control group. Additionally, numbers of patients are still quite low, the patient collective is heterogeneous, and the performed surgical procedures have a certain variety according to the underlying individual pathology. Another limitation is the missing of an objective evaluation of the patients foot abduction/adduction. This aspect should be part of the evaluation for a successful surgical reconstruction but was intentionally discarded due to the smaller reproducibility compared to the evaluation of the calcaneal pitch and Meary’s angle in the lateral radiograph. The main conclusion of this manuscript to primarily address the equinus position of the hindfoot permanently by corrective hindfoot arthrodesis still has to be confirmed by further studies with a long-time follow-up which are currently running at our institution.

## Conclusion

Surgical treatment of Charcot arthropathy remains a considerable challenge. Although new implants have been developed during the last years and various therapy algorithms have been proposed, the complication rates are still very high. Surgical correction of an equinus deformity of the hindfoot has been highlighted as a key goal for successful treatment.

Although surgical Achilles tendon lengthening was performed in this study, this procedure was not able to sufficiently impair development of complications in these patients surgically treated in a single-stage protocol. Multi-stage procedures include prolonged hospitalization times with risk for additional complications due to immobilization besides socioeconomic disadvantages.

**Fig. 7:**
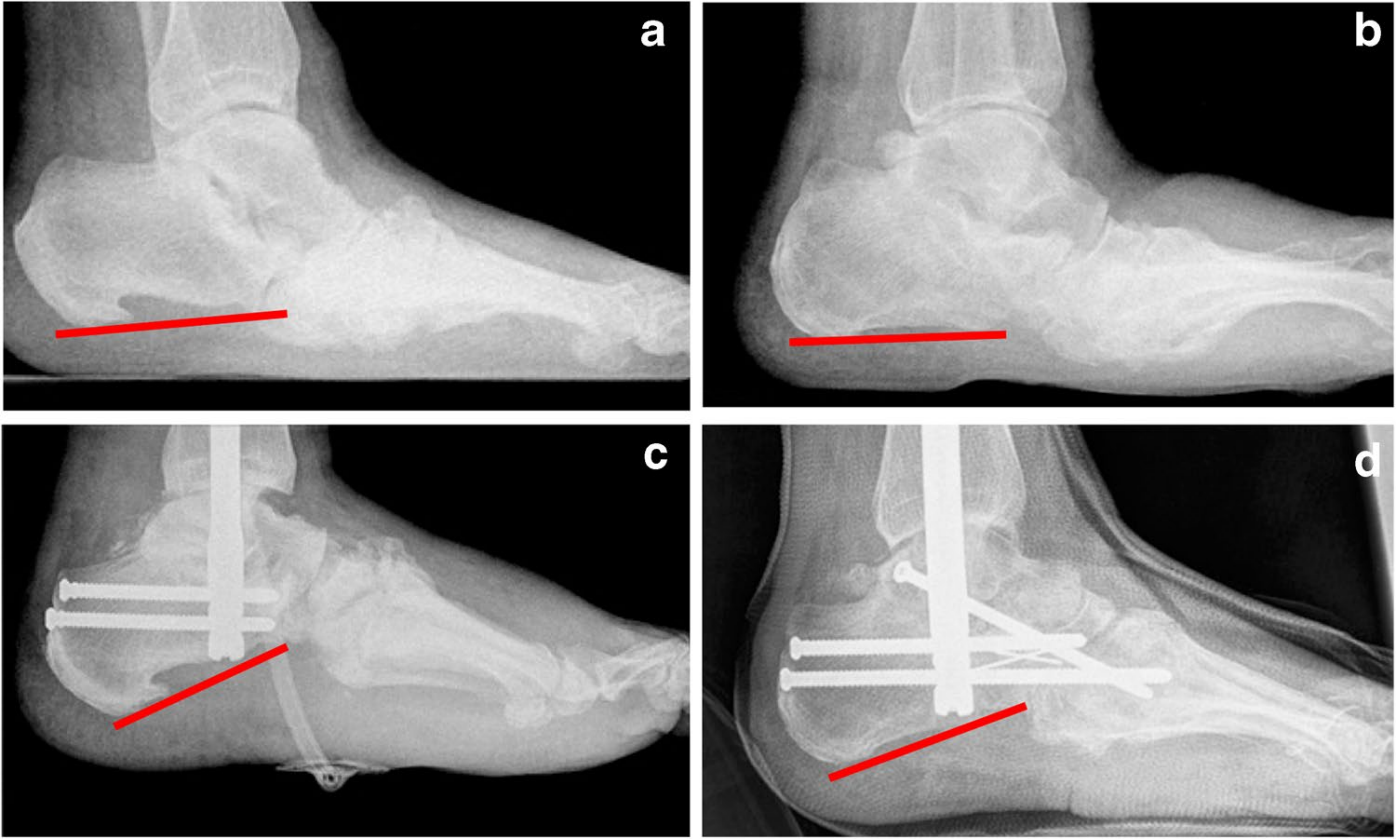
Examples of two cases treated with primary hindfoot arthrodesis in a 61-year-old (**a**) as well as a 72-year-old (**b**) patient with destructive Charcot arthropathy. Hereby, calcaneal pitch was corrected towards normal position with 23° (**c**) and 18° (**d**)

As a conclusion of the results of our study, we have changed our surgical regime towards a primary definitive correction of the pathological hindfoot position by corrective hindfoot arthrodesis and abandoned primary resection arthrodesis of the midfoot (Fig. 7). However, further studies comparing long-term outcomes need to be performed to be able to decide which treatment regime is actually preferable.

## Data Availability

Not applicable.
